# Transcriptome Analysis of the Scleractinian Coral *Stylophora pistillata*

**DOI:** 10.1371/journal.pone.0088615

**Published:** 2014-02-13

**Authors:** Sarit Karako-Lampert, Didier Zoccola, Mali Salmon-Divon, Mark Katzenellenbogen, Sylvie Tambutté, Anthony Bertucci, Ove Hoegh-Guldberg, Emeline Deleury, Denis Allemand, Oren Levy

**Affiliations:** 1 The Mina and Everard Goodman Faculty of Life Sciences, Bar-Ilan University, Ramat Gan, Israel; 2 Centre Scientifique de Monaco, Monaco, Monaco; 3 Department of Molecular Biology, Ariel University, Ariel, Israel; 4 Université de Nice-Sophia-Antipolis, UFR Sciences, Nice, France; 5 Australian Research Council Centre of Excellence for Coral Reef Studies, James Cook University, Townsville, Queensland, Australia; 6 Global Change Institute, The University of Queensland, St Lucia, Queensland, Australia; 7 Institut Sophia Agrobiotech INRA 1355, CNRS 7254, Sophia-Antipolis, France; King Abdullah University of Science and Technology, Saudi Arabia

## Abstract

The principal architects of coral reefs are the scleractinian corals; these species are divided in two major clades referred to as “robust” and “complex” corals. Although the molecular diversity of the “complex” clade has received considerable attention, with several expressed sequence tag (EST) libraries and a complete genome sequence having been constructed, the “robust” corals have received far less attention, despite the fact that robust corals have been prominent focal points for ecological and physiological studies. Filling this gap affords important opportunities to extend these studies and to improve our understanding of the differences between the two major clades. Here, we present an EST library from *Stylophora pistillata* (Esper 1797) and systematically analyze the assembled transcripts compared to putative homologs from the complete proteomes of six well-characterized metazoans: *Nematostella vectensis, Hydra magnipapillata*, *Caenorhabditis elegans, Drosophila melanogaster, Strongylocentrotus purpuratus*, *Ciona intestinalis* and *Homo sapiens*. Furthermore, comparative analyses of the *Stylophora pistillata* ESTs were performed against several Cnidaria from the Scleractinia, Actiniaria and Hydrozoa, as well as against other stony corals separately. Functional characterization of *S. pistillata* transcripts into KOG/COG categories and further description of Wnt and bone morphogenetic protein (BMP) signaling pathways showed that the assembled EST library provides sufficient data and coverage. These features of this new library suggest considerable opportunities for extending our understanding of the molecular and physiological behavior of “robust” corals.

## Introduction

Corals and sea anemones include some of the morphologically simplest metazoan animals. As members of the phylum Cnidaria, a sister group of the Bilateria, they have proven to be particularly significant for deriving information about the gene content of the common metazoan ancestor, the Ureumetazoan [1]. Cnidarians are characterized as having a single body axis with only two germ layers and two to three cell lineages, which gives rise to a handful of different cell types [2]. This profile suggests an underlying genetic simplicity and that cnidarian body plans are likely to be specified and patterned by a subset of the genes known among ‘higher’ animals. However, several recent studies [3]–[5] have challenged this idea. Analyses of the *Hydra magnipapillata*, *Nematostella vectensis* and *Acropora digitifera* genomes have revealed that cnidarians possess homologs of many of the developmental genes that control the axial specification of the Bilaterian body plan [3], [6]–[8]. Two examples are the Wnt and the BMP pathways.

The Wnt proteins are one of six families of signaling molecules that are responsible for the majority of developmental cell-cell interactions. They play important roles during vertebrate and invertebrate development and have the ability to activate different intracellular signaling pathways. In spite of the diverse roles of the Wnt’s across the Bilateria, Kusserow et al. [4] have recognized certain conserved expression patterns in *Nematostella* by identifying a near-complete Wnt complement. They identified 12 of the 13 known vertebrate and invertebrate members of the Wnt subfamilies. This number is greater than that found in *Hydra,* which have most likely lost two of the ancestral Wnt subfamilies [9]. In contrast, the Ecdysozoa are known to have lost half of the ancestral Wnt diversity. The available data on the Wnt family reveal that there was greater diversity in the common ancestor of higher animals compared to extant species, which indicates gene loss over the course of animal evolution [10]. Bone morphogenetic proteins (BMPs) are members of the transforming growth factor-β (TGF-β) superfamily of proteins, which includes TGF-βs, activins, and inhibins [11], and act as differentiation factors. BMP protein family members have been identified in a wide range of vertebrates (see [12] for review) as well as invertebrates such as nematode worms [13], flies [14], [15], sea urchins [16], mollusks [17] and cnidarians [18]–[20]. Although the TGF-β superfamily exists in all animals, it appears that BMP2/4 homologues [20] and BMP receptors [21] arose after the Porifera (sponge)/(cnidarian,+bilateral metazoa) split because BMP2/4 homologues are not found in sponges. Among Cnidaria, the BMP2/4 ortholog (bmp2/4-Am) is differentially expressed in the larval stage in *Acropora millepora* and is assumed to play a role in tissue differentiation and axis determination [19], [22].

**Figure 1:**
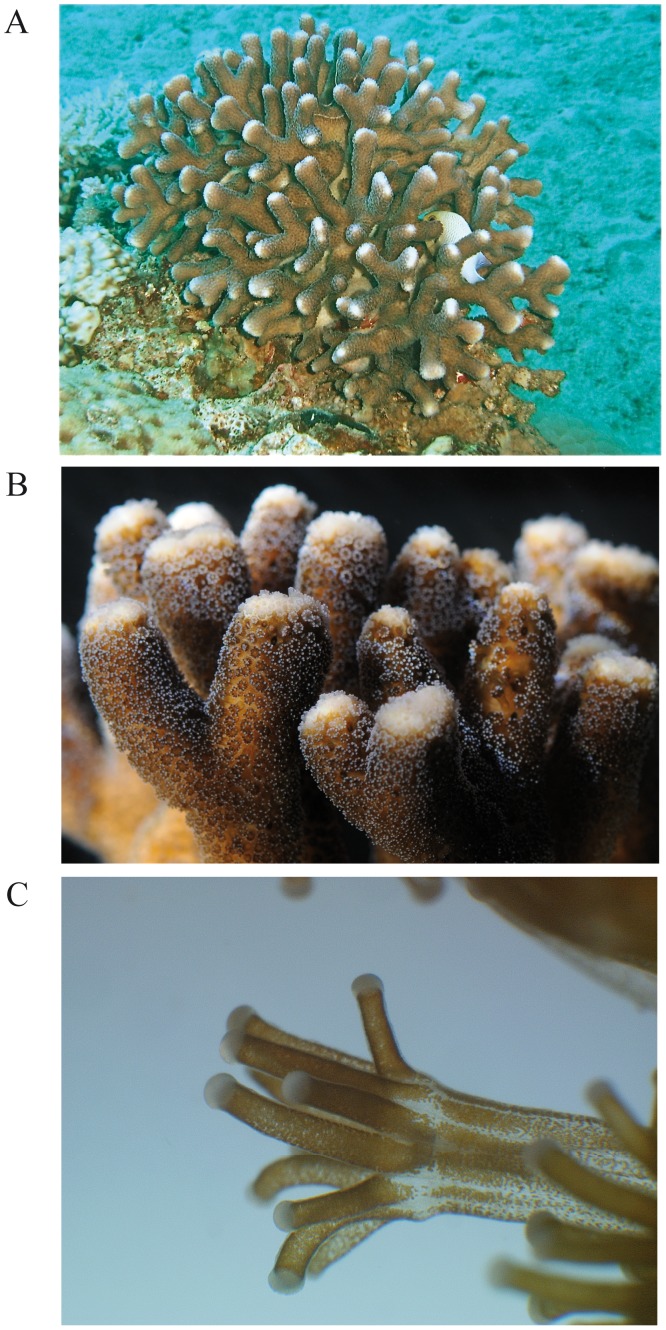
*Stylophora pistillata* (Esper, 1797) is a member of the “robust” lineage of stony corals. (A) A colony of *S. pistillata* growing in the Gulf of Eilat, Israel. (B) A colony in the aquarium at the Centre Scientifique de Monaco. (C) Magnification of a polyp in which the symbiotic dinoflagellates (*Symbiodinium* sp.) can be observed. (Credits-A: D. Zoccola; B &C: E. Tambutté/CSM).

Among scleractinians, several phylogenetic studies have shown that the Scleractinia are divided into two primary lineages: the “complex” and “robust” clades [23]–[27]. Acroporidae, which includes *Acropora*, belongs to the “complex” clade. As shown by Shinzato et al. [7], there is a divergence between the “complex” and “robust” clades, necessitating studying corals from both clades. Four EST libraries from “complex” species [3], [28]–[31] and two EST libraries of “robust” species [30]–[32] have been created to date. In this study, we performed EST sequencing of *Stylophora pistillata,* which is abundant in most coral reefs of the Indo-Pacific region (Fig. 1). This species has become a classic cnidarian model organism for studying symbiosis, physiology, cell biology, the calcification process and photosynthesis [33]–[44].

In the present study, 521,460 reads were generated from the coral holobiont, and were assembled as 15,052 contigs. We searched for putative coral protein homologs within a comprehensive non-redundant proteome database that included the complete genomes of six metazoan organisms: the cnidarians *Nematostella vectensis* (starlet sea anemone) and *Hydra magnipapillata*, the nematode *Caenorhabditis elegans* (worm), the arthropod *Drosophila melanogaster* (fruit fly), the echinoderm *Strongylocentrotus purpuratus* (purple sea urchin), the urochordate *Ciona intestinalis* (sea squirt) and the vertebrate *Homo sapiens* (human). Furthermore, comparative EST analyses were performed within the Cnidaria (Scleractinia, Actiniaria and Hydrozoa) and stony corals. Additionally, comparisons with the human proteome allowed us to delineate clusters of orthologous groups (COGs) and to focus on two developmental pathways (the Wnt and BMP pathways). This study provides a strong platform for further research on the molecular aspects of physiological and behavioral processes in corals.

## Methods

### Coral Sampling and Growth Conditions

Adult colonies of *Stylophora pistillata* were collected either from the field or from corals maintained in tanks for at least 20 years within the aquarium system of the Centre Scientifique de Monaco. The corals collected from the field (the Israeli Nature and National Parks Protection Authority approved the collection of corals in this study, permit No. 2011/38353) came from the Gulf of Aqaba in the Red Sea and were transferred after collection to tanks at the Marine Station of Eilat, Israel. The tanks were supplied continuously with seawater from the Red Sea. After an acclimation period of two weeks, colonies of *S. pistillata* were separated into different tanks that exposed the colonies to different environmental conditions, as described below. The cultured corals were maintained in a 300-liter aquarium supplied with seawater from the Mediterranean Sea (exchange rate 2% h^−1^) under controlled conditions, as follows: semi-open circuit, temperature of 26.0±0.2°C, salinity of 38.2‰, and light intensity of 175 µmol photons m^−2^ s^−1^ (using fluorescent tubes from Custom Sea Life®) under a 12∶12 h photoperiod. The corals were fed three times weekly with a mixture of *Artemia salina* nauplii, frozen adults of *Artemia salina*, and frozen krill.

**Figure 2:**
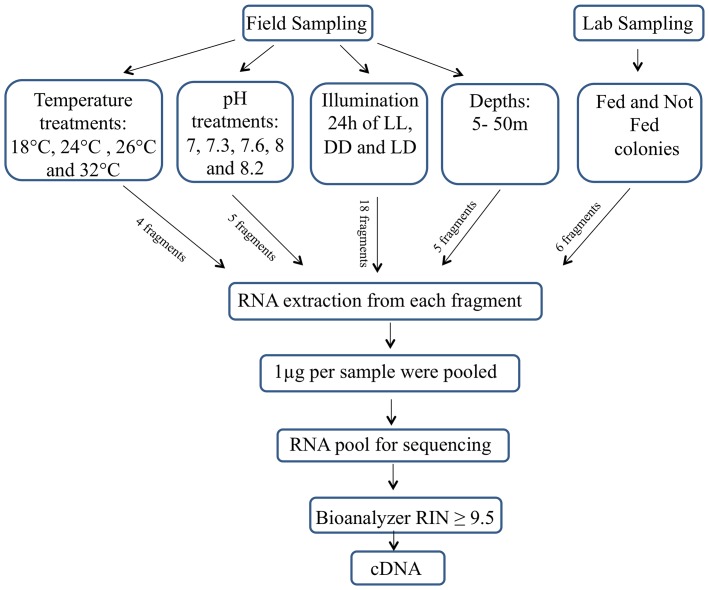
Flow chart of the experimental procedure. RNA was extracted from adult colonies of *Stylophora pistillata* from field and laboratory maintained colonies. The coral colonies were held under the presented treatments. LL – constant illumination; LD –12∶12 h light/dark; DD – constant dark.

Colonies from field and cultured sources were grown for two weeks under different conditions (Fig. 2) in order to maximize the expression of the greatest variety of genes. These conditions include different temperatures (18°C, 26°C and 32°C), different light/dark cycles, different pH levels (7 to 8.2), and either fed or not fed. Different field conditions were also included, with colonies being exposed to depths ranging from 5 to 50 meters. After exposure to different treatments, each colony was divided into fragments and snap-frozen in liquid nitrogen.

### RNA Extraction and cDNA Library Construction

Total RNA was isolated from each of the fragments from the different treatments described above using TRIzol (Invitrogen) according to manufacturer’s instructions. The quality of all of the RNA was checked using a Bioanalyzer (Agilent) RIN ≥9.5, and pools with the same amount of RNA were then created. The cDNA library was constructed using a Clontech SMARTer PCR cDNA synthesis kit and amplified using the Advantage 2 PCR kit according to the manufacturer's instructions. Subsequently, 2 µg of the amplified cDNA was normalized using the Trimmer kit (Evrogen) following the manufacturer's instructions and purified using the Qiaquick PCR purification kit (Qiagen). The normalized and non-normalized cDNA was sent to Roche for further analysis. Sequencing was performed using a 454 GS-Flx instrument according to the manufacturers' instructions (Roche). In order to obtain the abundant and rare transcripts, the library placed on the 454 plate was divided into two, half containing normalized cDNA, and the other half with non-normalized cDNA. The normalized and non-normalized cDNAs were sheared by sonication to produce short random fragments (300–400 bp) appropriate for 454 sequencing, and oligonucleotide adaptors were then ligated to the fragmented sequences. The 454 GS-Flx running plate was divided for internal comparison of normalized and non-normalized libraries (data not shown).

### Sequence, Data Assembly and Analysis

The sequences were submitted to Newbler assembler version 2.6 for de novo assembly of 454-sequenced EST libraries using the default parameters. The assembled sequences were first automatically annotated with the SwissProt databases and then with several species-specific databases using the BLAST program. The species utilized in this analysis are as follows: the sea anemones *Nematostella vectensis*, *Aiptasia pallida*, *Metridium senile*, and *Anemonia viridis*; the stony corals *Acropora millepora*, *Acropora palmata*, *Acropora digitifera, Montastraea faveolata*, and *Porites astreoides*; the hydrozoans *Clytia hemisphaerica* and *Hydra vulgaris*; and the protist *Symbiodinium* sp. The best matches obtained using the following blast parameters: e-value ≤1e-^10^, best-hit-overhang  = 0.1, best-hit-score-edge  = 0.1; in order to avoid random short hits. Pathway analysis was later performed by running a pairwise sequence search compared with the KEGG-curated set of human proteins [45].

### Phylogenetic Analyses

The alignments of all amino acid sequences were performed with the Multalin server [46] and phylogenetic relationships were investigated using Bayesian techniques as implemented in the computer program MrBayes v3.1.2 [47], starting from a random tree, generating 3,500,000 generations with sampling every 1000 generations, and with four chains in order to obtain the final (consensus) tree and to determine the posterior probabilities at the different nodes.

## Results

### EST Library Construction and Assembly

The normalized and non-normalized cDNA libraries constructed from *S. pistillata* holobiont RNA starting materials were based on an RNA pool collected from different environmental conditions to maximize the diversity of rarely expressed genes. Normalization of the library decreased the amounts of abundant transcripts and maximized the chances of finding new genes. We divided the 454 plate to run normalized and non-normalized cDNA libraries to obtain both abundant and rare transcripts. The two datasets were merged before assembly to generate a database of 523,533 sequenced reads. Assembly of these reads produced in 15,052 contigs with a mean length of 1,078 bp and N50 1,256 bp. These results are available from the NCBI and from (http://data.centrescientifique.mc/). Using BLAST searches against SwissProt database we were able to annotate 51% of the obtained sequences.

### Comparative Analysis

**Figure 3:**
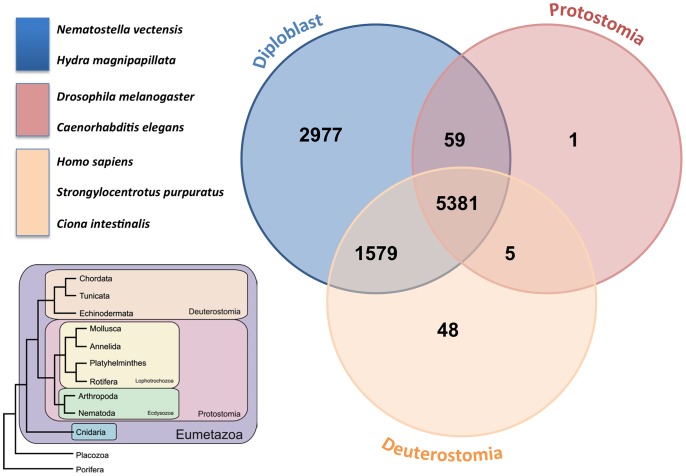
Venn diagram of transcript homologues from the stony coral *Stylophora pistillata* across taxonomic groups, including diploblasts (A. digitifera, *H. magnipapillata, N. vectensis,*); protostomes (*D. Melanogaster and C. elegans*); and deuterostomes (*S. Purpuratus, C. intestinalis and H. sapiens*).

*S. pistillata* transcript similarity searches were first performed against proteome libraries using blastX (see methods for specific parameters) (Table 1). At this level, 10,050 out of the 15,052 contigs (∼67%) exhibited a positive match to proteins from one or more species. As expected, most of the *S. pistillata* transcripts matched sequences from the coral *A. digitifera* [7] and *N. vectensis.* The human proteome ranked third in terms of the number of hits, followed by the third diploblast *Hydra magnipapillata*. The two protostomes (the fruit fly and nematode) presented the lowest numbers of hits and were ranked last. The general tendency of the homolog presence across lineages is even more apparent if we group *S. pistillata* homologs across taxonomic groups, as shown in Fig 3. Most of the coral transcripts (5,381) were found in all three groups followed by transcripts found specifically in at least one of the species belonging to the diploblast group, (2,977). Moreover, a large set of diploblast homologs was found among the deuterostome representatives (1,579), supporting the findings of previous work in cnidarians [3], [4], [6], [10] that has shown an unexpectedly high homology of diploblasts with deuterostomes in comparison to protostomes [48].

**Table 1:** The number of contigs with at least one valid hit obtained using blastX to search the *S. pistillata* EST library against various proteome libraries.

Proteome	Species	Number of proteins	Number of contigs
**DIPLOBLASTS**	*Hydra magnipapillata* (JGI)	32338	6084
	*Nematostella vectensis* (JGI)	27273	8228
	*Acropora digitifera* (Shinzato et al,2011)	23677	8868
**DEUTEROSTOMES**	*Homo sapiens* (Ensembl v.70)	104785	6377
	*Ciona intestinalis* (Ensembl)	17289	5578
	*S. purpuratus* (NCBI)	44303	5288
**PROTOSTOMES**	*Drosophila melanogaster* (Ensembl v.70)	26950	5123
	*Caenorhabditis elegans* (Ensembl v.70)	31234	4493

**Figure 4:**
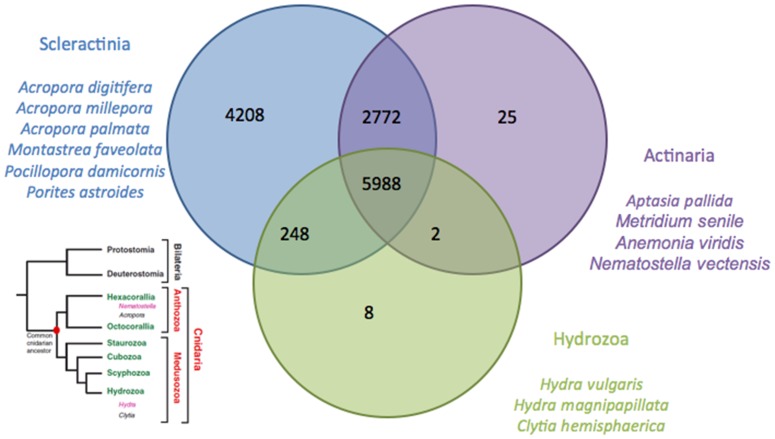
Venn diagram of transcript homologues from *Stylophora pistillata* across cnidarians (Scleractinia, Actiniaria, and Hydrozoa). The positions of the different groups are indicated in the left tree, obtained from Technau and Steele [63].

To study the differences between our EST library and those of the Cnidaria in general, we compared it to the available cnidarian EST libraries (see methods). Cross matches of *Stylophora* transcript homologs across Cnidaria (Table 2 and Fig. 4) showed 13,216 hits with stony corals, 8,787 with anemones and 6,246 with hydrozoans. Out of these, 5,988 were common to all three families.

**Table 2:** The number of contigs with at least one valid hit obtained from a blastN search using the EST library from *S. pistillata* as a query against various cnidarian EST libraries.

Groups	Species	Number of ESTs	Number of contigs
Stony corals (Cnidaria; Anthozoa;Hexacorallia; Scleractinia)	*Acropora digitifera* (Shinzato et al,2011)	36,780	9,822
	*Acropora millepora* (Moya et al 2012)	52,963	10,560
	*Acropora palmate* (NCBI)	43151	6,626
	*Montastraea faveolata* (NCBI)	33,206	6,000
	*Pocillopora damicornis*	72,890	11,661
	*Porites astreoides* (matzLAB)	92,142	8,755
`x`x`Sea anemones (Cnidaria; Anthozoa;Hexacorallia; Actiniaria)	*Aiptasia pallida* (NCBI)	19,404	3,193
	*Anemonia viridis* (NCBI)	49,667	5,419
	*Metridium senile (NCBI)*	29,471	4,259
	*Nematostella vectensis (JGI)*	164,163	7,842
Hydrozoans (Cnidaria; Hydrozoa;Hydroida)	*Clytia hemisphaerica (NCBI)*	85,991	4,066
	*Hydra vulgaris (NCBI)*	179,642	4,025
	*Hydra magnipapillata (JGI)*	146,429	5,244

### Functional Characterization

**Figure 5:**
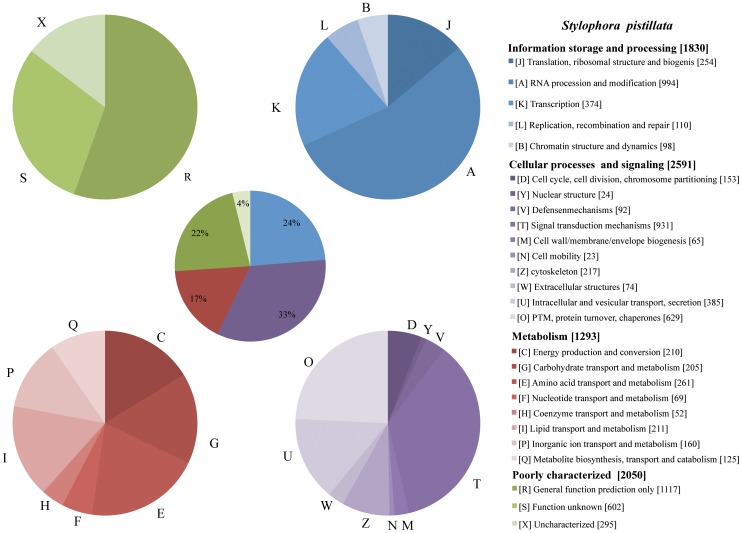
Functional characterization of *S. pistillata* transcripts. A total of 8,667 transcripts were classified into KOG/COG categories, giving rise to a total of 9,769 class assignments (some COGs belong to more than one class). The distribution among the functional classes is given in the central pie chart, with each super category slice broken down into separate pie charts in the corners (poorly characterized and uncharacterized functional categories [R and S] are combined with the uncharacterized category [X] in a separate pie chart at the top left corner). The overall class distribution follows those of other metazoan genomes, with the most abundant functions being signal transduction, protein turnover, translation and transcription.

**Figure 6:**
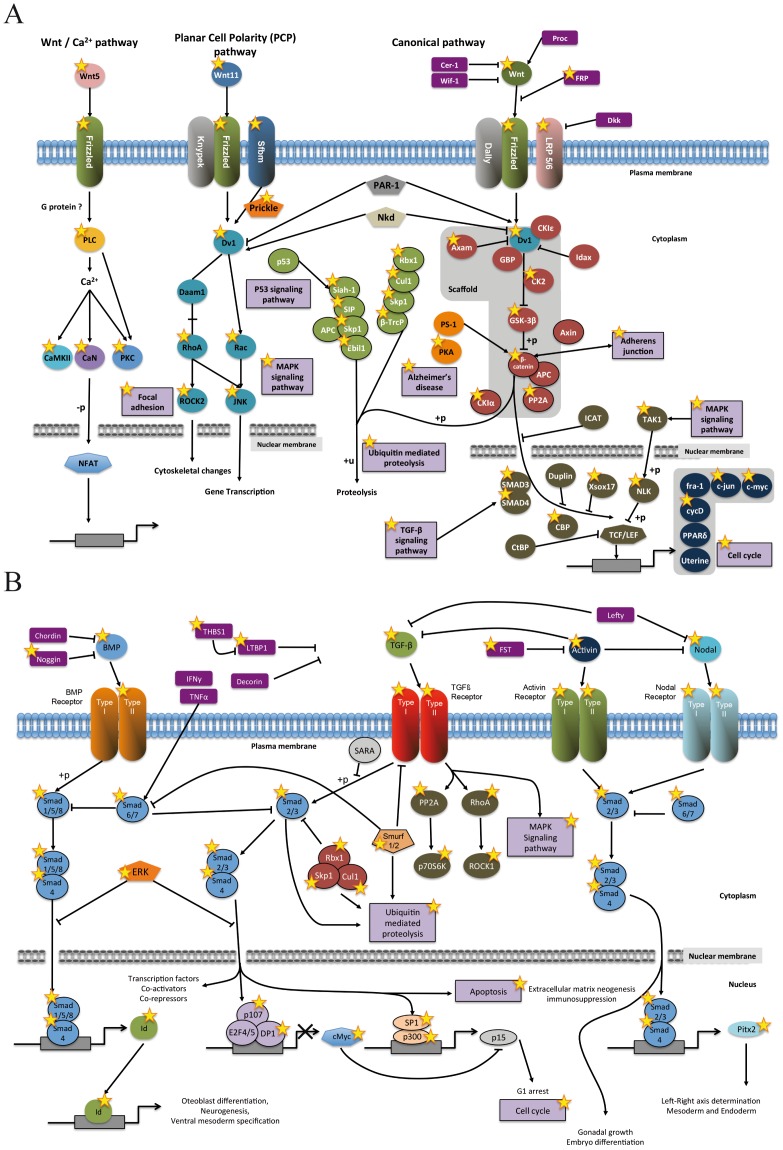
Human Wnt (A) and BMP (B) signaling pathways from KEGG pathways [**45**]. Sequences found in the *Stylophora pistillata* EST library based on sequence similarity with equivalent human homologues are labeled with a yellow star.

After the evolutionary comparisons of homology between related and non-related lineages were complete, we successfully classified 7,764 *S. pistillata* transcripts using the STRING database v.6.3 [49] and a stringent assignment process. The graphical distribution of functional classes is illustrated in Figure 5. When comparing functional data between *S. pistillata* transcripts and the human proteome, we observed the presence of equivalent functional elements. These elements are sufficient to reconstitute key processes in signaling and cell adhesion, which we chose to further explore by focusing on Wnt and BMP pathways (Fig. 6). The wingless (Wnt) and bone morphogenetic protein (BMP) pathways are essentially independent signaling mechanisms, although they often regulate similar biological processes. Using DAVID bioinformatics resources [50], we revealed the presence of equivalent functional elements in the Wnt and TGF-β signaling pathways (Fig. 6).

## Discussion

### EST Library Construction and Assembly

The transcriptome reported in this study comprises 15,052 sequences, which is less than the estimated number of genes in the cnidarian genomes described to date (32,338, 27,273 and 23,668 for *Hydra magnipapillata*, *Nematostella vectensis,* and *Acropora digitifera,* respectively). The raw reads were also assembled by MIRA 3.02 software [54] which produced a significantly higher number of contigs (48,075 ), however, a comparison between the two assemblies demonstrated that Newbler assembly was much less fragmented (data not shown), and hence was used for downstream analysis. We assume that the low number of contigs assembled is due to limited coverage, and that Newbler software produced an uncompleted albeit un-fragmented EST library.

### Comparative Analysis

The outcome of the cross matches from the Venn diagram shown in Figure 3 illustrates that the highest number of unique hits exists between *S. pistillata* and the stony corals, as expected. Actiniaria are also hexacorallia but are solitary animals and do not produce a skeleton. The number of unique cross matches between these two groups is lower, suggesting that biomineralization involves an important number of specific genes, as suggested by Shinzato et al. [7]. Finally, the lowest numbers of cross matches are obtained among the hydrozoans, which are more divergent (see the phylogenic tree in Fig. 4 obtained from Technau and Steele 2011), solitary and freshwater animals, which presumably accounts for part of the greater difference in the transcriptome.

It should be noted here that the cDNA library was constructed from holobiont tissue; hence, both zooxanthellae and host sequences are present in our library. Most coral libraries are constructed from larvae, which do not contain symbionts, with *P. damicornis* and *P. astreoides* being exceptions. However, a blastX comparison with *Symbiodinium* EST libraries [51], [52] downloaded from NCBI shows that few sequences mapped exclusively to the *Symbiodinium* genome and not to the coral *A. digitifera* (375 out of 15,052, i.e., ∼ 2.5% of sequences) illustrating that most of the contigs belong to the coral itself.

**Figure 7:**
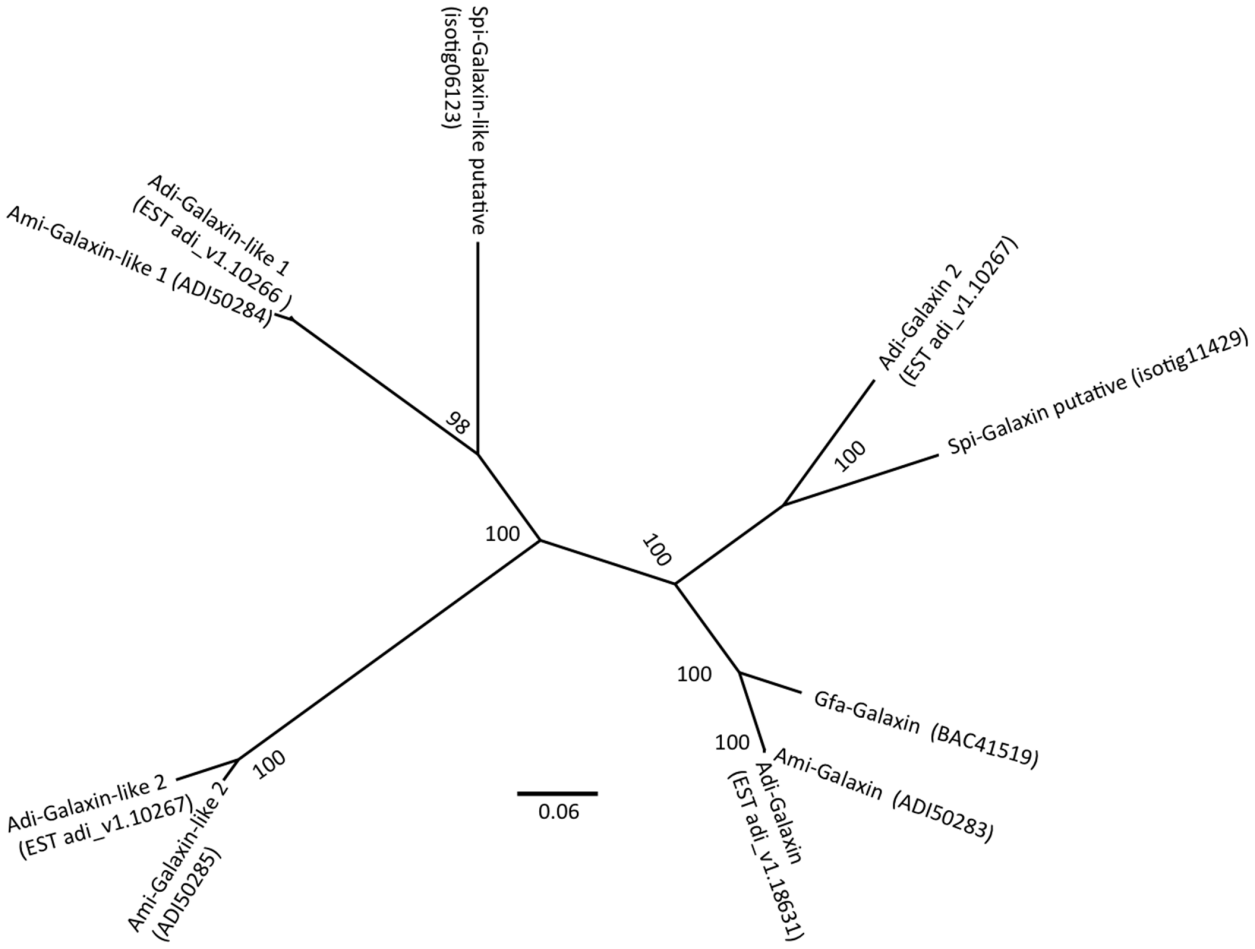
Galaxin phylogenetic tree. Galaxin and Galaxin-like sequences from different species: Spi-*Stylophora pistillata*, Adi-*Acropora digitifera*, Ami-*Acropora millepora*, Gfa- *Galaxea fascicularis* were used for phylogenetic analysis. The tree was constructed using Mrbayes program [46].

Among *Stylophora* ESTs, there are sequences coding for a specific organic matrix protein that provides the skeleton shape of the coral. The only protein to have been fully characterized from the calcifying matrix of scleractinian corals is galaxin, which was originally identified from the coral *Galaxea fascicularis* [53]. Based on similarity searches performed in an EST library from *Acropora millepora* [3], [54], Reyes-Bermudez et al. [55] reported the sequences of three genes encoding galaxin-related molecules and their expression patterns during settlement and metamorphosis. A fourth galaxin protein, galaxin 2, has been characterized by Shinzato et al. [7], based on the sequencing of *Acropora digitifera*. blastX and blastn searches of the galaxin sequences against the *S. pistillata* EST library identified two ESTs with strong similarity. A phylogenetic tree generated by MrBayes program classified these ESTs to galaxin 2 and galaxin-like 1 (Figure 7). No homolog for Amgalaxin-like 2 was found, probably since its expression is restricted to post-settlement polyps and is not observed in adults [56]. The presence of an Amgalaxin-like 1 homolog in an adult expression library is surprising, as Reyes-Bermudez et al. [55] showed that its expression remains restricted to the settlement and metamorphosis phases. Though, a species-specificity in galaxin-like diversity cannot be excluded. The availability of new coral genomes, from both robust and complex clades, is then a key feature in understanding the role and the impact of matrix proteins in coral calcification.

### Functional Characterization

Concerning the functional classification, half the transcripts show similarity to sequences in the KEGG database (51%). The distribution of functional classes is consistent with those in both the human and fruit fly complete proteomes [56], with the most abundant categories being RNA processing and modification (A) signal transduction (T), protein turnover (O), and translation (J), indicating that the coverage of the EST library presented here was adequate (Figure 5). We decide to focus on the Wnt and BMP signal transduction pathways because of their importance in coral development [4]. These two pathways do not share any major components and can function independently of each other, which is accomplished by means of different ligands, different receptors and different cytoplasmic and nuclear signal transducers. Most of the proteins found in humans are also present in corals. However, some of them are missing in the EST data obtained in this study due to 1) the technical methodology applied (for example, the chordin gene in the BMP pathway, absent from our EST library, is present in other cnidarian species [57], [58] and was found in a new library constructed using genome sequencing (in prep.) and/or 2) the absence of these genes in Cnidaria in cases where the genes appeared after the coelomata split, as observed for the secreted cytokines IFN gamma [personal data].

Wnt and BMP play roles in vertebrate biomineralization in addition to their roles in development. Studies have shown that both BMP and Wnt pathways are involved in osteogenesis [59], [60]. Furthermore, in adult coral, BMP expression is restricted to the skeletogenic ectoderm [42], and it has been suggested that BMP might be an organic matrix protein [61]. Although biomineralization and the nature of the calcifying organic matrix have been extensively studied in other taxa, such as mollusks [62], there have been relatively few corresponding studies of these elements in stony corals. We show here that some signaling pathways are conserved in *S. pistillata* providing new tools to understand the mechanisms of biomineralization. For instance, the study of BMP receptors and their location in polyps would help us to better understand the role of BMPs in adult corals.

## Conclusions

Databases of expressed sequence tags have become a widely used tool in fundamental biological research, primarily due to the considerable resources now available for searching novel gene sequences. Our study of the ubiquitous robust coral *Stylophora pistillata* provides an opportunity to explore a series of questions concerning comparative and functional genomics and the evolutionary relationships among the stony corals and invertebrates in general. In conducting this study, we have been able to investigate the ancestry of several important metazoan genes. The basal phylogenetic position of the anthozoans and the surprising extent to which key genes appear to have been conserved between Cnidaria and chordates implies that further research in corals is likely to provide unique perspectives on common molecular principles of animal development. Most of our knowledge on the physiology of coral calcification comes from studies in *Stylophora pistillata*, the present EST library will constitute a valuable tool for determining the molecular mechanisms underlying coral growth and development and the molecular targets of environmental disturbances such as global warming and ocean acidification [61].

## References

[1] Ball EE, Hayward DC, Saint R, Miller DJ (2004) A simple plan–cnidarians and the origins of developmental mechanisms. Nature reviews Genetics 5: 567–577. 10.1038/nrg1402 15266339

[2] Bode HR (1996) The interstitial cell lineage of hydra: a stem cell system that arose early in evolution. Journal of cell science 109 (Pt 6): 1155–1164. 10.1242/jcs.109.6.1155 8799806

[3] Kortschak RD, Samuel G, Saint R, Miller DJ (2003) EST analysis of the cnidarian Acropora millepora reveals extensive gene loss and rapid sequence divergence in the model invertebrates. Current biology : CB 13: 2190–2195. 14680636 10.1016/j.cub.2003.11.030

[4] Kusserow A, Pang K, Sturm C, Hrouda M, Lentfer J, et al (2005) Unexpected complexity of the Wnt gene family in a sea anemone. Nature 433: 156–160. 15650739 10.1038/nature03158

[5] Steele RE, Stover NA, Sakaguchi M (1999) Appearance and disappearance of Syk family protein-tyrosine kinase genes during metazoan evolution. Gene 239: 91–97. 10571038 10.1016/s0378-1119(99)00373-x

[6] Matus DQ, Pang K, Marlow H, Dunn CW, Thomsen GH, et al (2006) Molecular evidence for deep evolutionary roots of bilaterality in animal development. Proceedings of the National Academy of Sciences of the United States of America 103: 11195–11200. 16837574 10.1073/pnas.0601257103 PMC1544064

[7] Shinzato C, Shoguchi E, Kawashima T, Hamada M, Hisata K, et al. (2011) Using the Acropora digitifera genome to understand coral responses to environmental change. Nature. 10.1038/nature10249 21785439

[8] Srivastava M, Simakov O, Chapman J, Fahey B, Gauthier ME, et al (2010) The Amphimedon queenslandica genome and the evolution of animal complexity. Nature 466: 720–726. 20686567 10.1038/nature09201 PMC3130542

[9] Lengfeld T, Watanabe H, Simakov O, Lindgens D, Gee L, et al (2009) Multiple Wnts are involved in Hydra organizer formation and regeneration. Developmental biology 330: 186–199. 19217898 10.1016/j.ydbio.2009.02.004

[10] Miller DJ, Ball EE, Technau U (2005) Cnidarians and ancestral genetic complexity in the animal kingdom. Trends in genetics : TIG 21: 536–539. 16098631 10.1016/j.tig.2005.08.002

[11] Wozney JM, Rosen V, Celeste AJ, Mitsock LM, Whitters MJ, et al (1988) Novel regulators of bone formation: molecular clones and activities. Science 242: 1528–1534. 3201241 10.1126/science.3201241

[12] Wozney JM (2002) Overview of bone Morphogenetic Proteins. Spine 27: S2–S8. 12205411 10.1097/00007632-200208151-00002

[13] Suzuki Y, Yandell MD, Roy PJ, Krishna S, Savage-Dunn C, et al (1999) A BMP homolog acts as a dose-dependent regulator of body size and male tail patterning in Caenorhabditis elegans. Development 126: 241–250. 9847238 10.1242/dev.126.2.241

[14] Padgett RW, St Johnston RD, Gelbart WM (1987) A transcript from a Drosophila pattern gene predicts a protein homologous to the transforming growth factor-beta family. Nature 325: 81–84. 3467201 10.1038/325081a0

[15] Wharton KA, Thomsen GH, Gelbart WM (1991) Drosophila 60A gene, another transforming growth factor beta family member, is closely related to human bone morphogenetic proteins. Proc Natl Acad Sci U S A 88: 9214–9218. 1924384 10.1073/pnas.88.20.9214 PMC52684

[16] Hwang SL, Chen CA, Chen C (1999) Sea urchin TgBMP2/4 gene encoding a bone morphogenetic protein closely related to vertebrate BMP2 and BMP4 with maximal expression at the later stages of embryonic development. Biochem Biophys Res Commun 258: 457–463. 10329409 10.1006/bbrc.1999.0663

[17] Nederbragt AJ, van Loon AE, Dictus WJ (2002) Expression of Patella vulgata orthologs of engrailed and dpp-BMP2/4 in adjacent domains during molluscan shell development suggests a conserved compartment boundary mechanism. Dev Biol 246: 341–355. 12051820 10.1006/dbio.2002.0653

[18] Lelong C, Mathieu M, Favrel P (2001) Identification of new bone morphogenetic protein-related members in invertebrates. Biochimie 83: 423–426. 11368850 10.1016/s0300-9084(01)01260-3

[19] Hayward DC, Samuel G, Pontynen PC, Catmull J, Saint R, et al (2002) Localized expression of a dpp/BMP2/4 ortholog in a coral embryo. Proc Natl Acad Sci U S A 99: 8106–8111. 12048233 10.1073/pnas.112021499 PMC123028

[20] Hwang SL, Chen CA, Peng M, Chen C (2003) Evolutionary conservation of the bone morphogenetic protein 2/4 gene between diploblastic and triploblastic metazoans. Zoological Studies 42: 227–234.

[21] Suga H, Ono K, Miyata T (1999) Multiple TGF-beta receptor related genes in sponge and ancient gene duplications before the parazoan-eumetazoan split. FEBS Lett 453: 346–350. 10405173 10.1016/s0014-5793(99)00749-8

[22] Finnerty JR, Pang K, Burton P, Paulson D, Martindale MQ (2004) Origins of bilateral symmetry: Hox and dpp expression in a sea anemone. Science 304: 1335–1337. 15131263 10.1126/science.1091946

[23] Fukami H, Chen CA, Budd AF, Collins A, Wallace C, et al (2008) Mitochondrial and nuclear genes suggest that stony corals are monophyletic but most families of stony corals are not (Order Scleractinia, Class Anthozoa, Phylum Cnidaria). PloS one 3: e3222. 18795098 10.1371/journal.pone.0003222 PMC2528942

[24] Kerr AM (2005) Molecular and morphological supertree of stony corals (Anthozoa: Scleractinia) using matrix representation parsimony. Biological reviews of the Cambridge Philosophical Society 80: 543–558. 16221328 10.1017/S1464793105006780

[25] Le Goff-Vitry MC, Rogers AD, Baglow D (2004) A deep-sea slant on the molecular phylogeny of the Scleractinia. Mol Phylogenet Evol 30: 167–177. 15022767 10.1016/s1055-7903(03)00162-3

[26] Romano SL, Cairns SD (2000) Molecular phylogenetic hypotheses for the evolution of scleractinian corals. Bull Mar Sci 67: 1043–1068.

[27] Romano SL, Palumbi SR (1997) Molecular evolution of a portion of the mitochondrial 16S ribosomal gene region in scleractinian corals. J Mol Evol 45: 397–411. 9321419 10.1007/pl00006245

[28] Iguchi A, Shinzato C, Foret S, Miller DJ (2011) Identification of Fast-Evolving Genes in the Scleractinian Coral Acropora Using Comparative EST Analysis. PLoS One 6: e20140. 21701682 10.1371/journal.pone.0020140 PMC3119059

[29] Meyer E, Aglyamova GV, Wang S, Buchanan-Carter J, Abrego D, et al (2009) Sequencing and de novo analysis of a coral larval transcriptome using 454 GSFLx. BMC Genomics 10: 1–18. 19435504 10.1186/1471-2164-10-219 PMC2689275

[30] Schwarz JA, Brokstein PB, Voolstra C, Terry AY, Manohar CF, et al (2008) Coral life history and symbiosis: functional genomic resources for two reef building Caribbean corals, Acropora palmata and Montastraea faveolata. BMC Genomics 9: 97. 18298846 10.1186/1471-2164-9-97 PMC2291459

[31] Voolstra CR, Sunagawa S, Matz MV, Bayer T, Aranda M, et al (2011) Rapid evolution of coral proteins responsible for interaction with the environment. PLoS One 6: e20392. 21633702 10.1371/journal.pone.0020392 PMC3102110

[32] Traylor-Knowles N, Granger BR, Lubinski TJ, Parikh JR, Garamszegi S, et al (2011) Production of a reference transcriptome and transcriptomic database (PocilloporaBase) for the cauliflower coral, Pocillopora damicornis. BMC Genomics 12: 585. 22126435 10.1186/1471-2164-12-585 PMC3339375

[33] Allemand D, Tambutt EE, Girard JP, Jaubert J (1998) Organic matrix synthesis in the scleractinian coral stylophora pistillata: role in biomineralization and potential target of the organotin tributyltin. The Journal of experimental biology 201 (Pt 13): 2001–2009. 10.1242/jeb.201.13.2001 9622572

[34] Gattuso JP, Allemand D, Frankignoulle M (1999) Photosynthesis and calcification at cellular, organismal and community levels in coral reefs: A review on interactions and control by carbonate chemistry. Amer Zool 36.

[35] Loya Y (2000) Hommage to Stylophora pistillata: a significant coral species in Red Sea coral reef research. Plenary lecture, The 9th International Coral Reef Symposium, Bali, Indonesia. Darwin Medal.

[36] Muscatine L, Tambutte E, Allemand D (1997) Morphology of coral desmocytes, cells that anchor the calicoblastic epithelium to the skeleton. Coral Reefs 16: 205–213.

[37] Puverel S, Tambutte E, Zoccola D, Domart-Coulon I, Bouchot A, et al (2005) Antibodies against the organic matrix in scleractinians: a new tool to study coral biomineralization. Coral Reefs 24: 149–156.

[38] Rinkevich B, Loya Y (1984) Does light enhance calcification in hermatypic corals? Mar Biol 80: 1–6.

[39] Tambutté Eacute, Allemand D, Mueller E, Jaubert J (1996) A compartmental approach to the mechanism of calcification in hermatypic corals. The Journal of experimental biology 199: 1029–1041. 9318837 10.1242/jeb.199.5.1029

[40] Tambutté E, Allemand D, Bourge I, Gattuso J-P, Jaubert J (1995) An improved 45Ca protocol for investigating physiological mechanisms in coral calcification. Mar Biol 122: 453–459.

[41] Yamashiro H (1995) The effects of HEBP, an inhibitor of mineral deposition, upon photosynthesis and calcification in the scleractinian coral, Stylophora pistillata. J Exp Mar Bio Ecol 191: 57–63.

[42] Zoccola D, Moya A, Beranger GE, Tambutte E, Allemand D, et al (2009) Specific expression of BMP2/4 ortholog in biomineralizing tissues of corals and action on mouse BMP receptor. Marine biotechnology 11: 260–269. 18795368 10.1007/s10126-008-9141-6

[43] Zoccola D, Tambutte E, Kulhanek E, Puverel S, Scimeca JC, et al (2004) Molecular cloning and localization of a PMCA P-type calcium ATPase from the coral Stylophora pistillata. Biochimica et biophysica acta 1663: 117–126. 15157614 10.1016/j.bbamem.2004.02.010

[44] Zoccola D, Tambutte E, Senegas-Balas F, Michiels JF, Failla JP, et al (1999) Cloning of a calcium channel alpha1 subunit from the reef-building coral, Stylophora pistillata. Gene 227: 157–167. 10023047 10.1016/s0378-1119(98)00602-7

[45] Kanehisa M, Araki M, Goto S, Hattori M, Hirakawa M, et al (2008) KEGG for linking genomes to life and the environment. Nucleic acids research 36: D480–484. 18077471 10.1093/nar/gkm882 PMC2238879

[46] Corpet F (1988) Multiple sequence alignment with hierarchical clustering. Nucleic Acids Res 16: 10881–10890. 2849754 10.1093/nar/16.22.10881 PMC338945

[47] Huelsenbeck JP, Ronquist F (2001) MRBAYES: Bayesian inference of phylogenetic trees. Bioinformatics 17: 754–755. 11524383 10.1093/bioinformatics/17.8.754

[48] Ball EE, Hayward DC, Catmull J, Reece-Hoyes JS, Hislop NR, et al. Molecular control of development in the reef coral, Acropora millepora. Proceedings of the 9th International Coral Reef Symposium (Bali, Indonesia), vol. 1; 2002; Jakarta. Indonesian Institute of Sciences. 395–402.

[49] von Mering C, Huynen M, Jaeggi D, Schmidt S, Bork P, et al (2003) STRING: a database of predicted functional associations between proteins. Nucleic acids research 31: 258–261. 12519996 10.1093/nar/gkg034 PMC165481

[50] Huang da W, Sherman BT, Lempicki RA (2009) Systematic and integrative analysis of large gene lists using DAVID bioinformatics resources. Nat Protoc 4: 44–57. 19131956 10.1038/nprot.2008.211

[51] Leggat W, Hoegh-Guldberg O, Dove S, Yellowlees D (2007) Analysis of an est library from the dinoflagellate (*Symbiodinium* sp.) symbiont of reef-building corals. J Phycol 43: 1010–1021.

[52] Bayer T, Aranda M, Sunagawa S, Yum LK, Desalvo MK, et al (2012) Symbiodinium transcriptomes: genome insights into the dinoflagellate symbionts of reef-building corals. PLoS One 7: e35269. 22529998 10.1371/journal.pone.0035269 PMC3329448

[53] Fukuda I, Ooki S, Fujita T, Murayama E, Nagasawa H, et al (2003) Molecular cloning of a cDNA encoding a soluble protein in the coral exoskeleton. Biochem Biophys Res Comm 304: 11–17. 12705876 10.1016/s0006-291x(03)00527-8

[54] Grasso LC, Maindonald J, Rudd S, Hayward DC, Saint R, et al (2008) Microarray analysis identifies candidates genes for key roles in coral development. BMC Genom 9: 540. 10.1186/1471-2164-9-540 PMC2629781 19014561

[55] Reyes-Bermudez A, Lin Z, Hayward DC, Miller DJ, Ball EE (2009) Differential expression of three galaxin-related genes during settlement and metamorphosis in the scleractinian coral Acropora millepora. BMC Evol Biol 9: 1–29. 19638240 10.1186/1471-2148-9-178 PMC2726143

[56] Tatusov RL, Fedorova ND, Jackson JD, Jacobs AR, Kiryutin B, et al (2003) The COG database: an updated version includes eukaryotes. BMC bioinformatics 4: 41. 12969510 10.1186/1471-2105-4-41 PMC222959

[57] Saina M, Genikhovich G, Renfer E, Technau U (2009) BMPs and chordin regulate patterning of the directive axis in a sea anemone. Proc Natl Acad Sci U S A 106: 18592–18597. 19833871 10.1073/pnas.0900151106 PMC2773963

[58] Rentzsch F, Anton R, Saina M, Hammerschmidt M, Holstein TW, et al (2006) Asymmetric expression of the BMP antagonists chordin and gremlin in the sea anemone Nematostella vectensis: implications for the evolution of axial patterning. Dev Biol 296: 375–387. 16828077 10.1016/j.ydbio.2006.06.003

[59] Rodda SJ, McMahon AP (2006) Distinct roles for Hedgehog and canonical Wnt signaling in specification, differentiation and maintenance of osteoblast progenitors. Development 133: 3231–3244. 16854976 10.1242/dev.02480

[60] Canalis E, Economides AN, Gazzerro E (2003) Bone Morphogenetic Proteins, Their Antagonists, and the Skeleton. Endocr Rev 24: 218–235. 12700180 10.1210/er.2002-0023

[61] Weis V, Allemand D (2009) What determines Coral Health? Science 324: 1153–1155. 19478172 10.1126/science.1172540

[62] Marin F, Luquet G, Marie B, Medakovic D (2008) Molluscan Shell Proteins: Primary Structure, Origin, and Evolution. Curr Top Dev Biol 80: 209–276. 17950376 10.1016/S0070-2153(07)80006-8

[63] Technau U, Steele RE (2011) Evolutionary crossroads in developmental biology: Cnidaria. Development 138: 1447–1458. 21389047 10.1242/dev.048959 PMC3062418

